# Antidepressant treatment in pregnancy: a Danish registry linkage study in pregnant women with pre-existing obsessive‐compulsive disorder

**DOI:** 10.1038/s41398-023-02516-0

**Published:** 2023-06-23

**Authors:** Nhung T. H. Trinh, Birgitte Dige Semark, Trine Munk-Olsen, Xiaoqin Liu, Suraj Bahadur Thapa, Zeynep Yilmaz, Liselotte Vogdrup Petersen, Angela Lupattelli

**Affiliations:** 1 PharmacoEpidemiology and Drug Safety Research Group, Department of Pharmacy, University of Oslo, Oslo, Norway; 2 NCRR-The National Centre for Register-based Research, Aarhus University, Aarhus, Denmark; 3 Centre for Integrated Register-based Research, CIRRAU, Aarhus University, Aarhus, Denmark; 4 Department of Clinical Research, University of Southern Denmark, Aarhus, Denmark; 5 Division of Mental Health and Addiction, Oslo University Hospital, Oslo, Norway; 6 Division of Mental Health and Addiction, Institute of Clinical Medicine, University of Oslo, Oslo, Norway; 7 Department of Biomedicine, Aarhus University, Aarhus, Denmark; 8 Department of Medical Epidemiology and Biostatistics, Karolinska Institutet, Stockholm, Sweden; 9 Department of Psychiatry, University of North Carolina at Chapel Hill, Chapel Hill, NC USA

**Keywords:** Psychiatric disorders, Scientific community

## Abstract

The association between antidepressant continuation during pregnancy and postpartum mental health in women with obsessive-compulsive disorder (OCD) is uncertain. We identified 1317 women with live-birth singleton pregnancies and having outpatient/inpatient visits for OCD in the 4 years pre-pregnancy from the Danish registries. We defined three groups based on antidepressant prescriptions filled in the 2 years before pregnancy to delivery: (i) unexposed (*n* = 449); (ii) discontinuers (*n* = 346), i.e., with pre-pregnancy antidepressant fills only; (iii) continuers (*n* = 522), i.e., with antidepressant fills before and during pregnancy. We estimated crude and propensity score weighted hazard ratio (HRs) of postpartum visit for OCD and mood/anxiety disorders using Cox proportional hazard models. In weighted analyses, we found no difference in the probability of a postpartum visit for OCD or MADs with antidepressant continuation compared to unexposed and discontinuers. The likelihood of a postpartum OCD visit was higher in pregnancies having only one prescription fill during pregnancy compared to unexposed (HR = 3.44, 95% CI: 1.24, 9.54) or discontinuers (HR = 2.49, 95% CI: 0.91, 6.83). Continuers in pregnancy without antidepressant fill in the first three months postpartum had higher probability for postpartum visit for mood/anxiety disorders compared to discontinuers (HR = 3.84, 95% CI: 1.49, 9.92). Among pregnant women with pre-existing OCD, we found similar probabilities of a postpartum visit for OCD or mood/anxiety disorders in antidepressant continuers compared to unexposed and discontinuers. Continuers with a single prescription fill during pregnancy or no fill postpartum may have higher risks for these outcomes. Our findings highlight the importance of continuity of treatment throughout the perinatal period.

## Introduction

Obsessive‐compulsive disorder (OCD) is a psychiatric disorder characterized by the presence of intrusive thoughts (obsessions) and ritualistic behaviour (compulsions) [1]. OCD causes significant distress to patients, impairing both work and social functioning [2, 3]. The perinatal period appears to be a time of high risk for the onset, relapse or exacerbation of OCD [4]. A longitudinal study following pregnant women until 9 months postpartum found a high prevalence of OCD during pregnancy (average prenatal point estimate = 2.9%), and the cumulative incidence of new OCD diagnoses was estimated at 9% by 6 months postpartum [5]. Postpartum OCD symptoms may consist of obsessional intrusive thoughts concerning contamination or aggression, leading to excessive overprotection or avoidance of the child or avoidance of the feared situation (e.g., harming the newborn) [1, 6]. Untreated OCD can negatively impact mother-infant bonding, care of the infant, mother’s functional behaviour and family well-being [7].

Psychotherapy, preferably cognitive behavioural therapy (CBT) with exposure and response prevention (ERP), remains the first-line treatment in patients with mild to moderate OCD symptoms [1]. Lately, an intensive psychotherapeutic treatment known as the Bergen 4-day treatment (B4DT) has been found to be effective for OCD [1, 8]. However, if symptoms are severe, uncontrolled, or include comorbid depression, pharmacotherapy with serotonergic antidepressants is recommended, preferably in combination with CBT [1, 8].

Existing studies suggest a high prevalence of antidepressant discontinuation when women enter the course of pregnancy, regardless of the underlying condition [9]. This pattern is likely due to concerns about child risks associated with medication exposure in utero [10] as per today there is a lack of clear guidelines so that clinicians have to take individual decisions for their patients. However, the psychiatric disorder itself, especially if sub-optimally treated, may jeopardize maternal-child health [11]. Because the postpartum represents a vulnerable period for women with OCD, understanding the role of antidepressant continuation in pregnancy on postpartum OCD symptoms and exacerbation is clinically relevant. Yet, evidence on potential benefits and risks associated with antidepressant continuation for both mother and child is lacking for OCD as most studies, to date, have focused on other conditions such as depression and anxiety [12].

In a population of pregnant women with pre-existing OCD, we examined the association of continued antidepressant treatment in pregnancy with specialist outpatient/inpatient visit for OCD from birth to 1 year postpartum, relative to discontinuation before pregnancy. Because of the high comorbidity of OCD with other psychiatric conditions [13], we also examined postpartum episodes of mood and anxiety disorders.

## Methods

### Study population

We conducted a cohort study utilising population-based linked data from multiple Danish national health registries using the Civil Registration number: the Danish Civil Registration System, the Danish Medical Birth Registry, the Danish National Patient Register, the Danish National Prescription Register, and the Danish Psychiatric Central Research Register. The description of these registries is given in detail elsewhere [14–17]. In brief: (1) the Danish Medical Birth Registry includes compulsory medical records on all live births and stillbirths since 1973 [15]; (2) the Danish National Patient Register includes admission records to hospitals since 1977 and outpatient specialist health care since 1995; (3) the Danish National Prescription Registry captures records on all prescriptions dispensed at community pharmacies in Denmark since 1995 [16]; and (4) the Danish Psychiatric Central Research Register includes records on all inpatient psychiatric treatment in Denmark since 1969 and outpatient since 1995 [17]. All diagnoses were coded according to the International Classification of Disease, 10th version (ICD-10) since 1994.

**Fig. 1:**
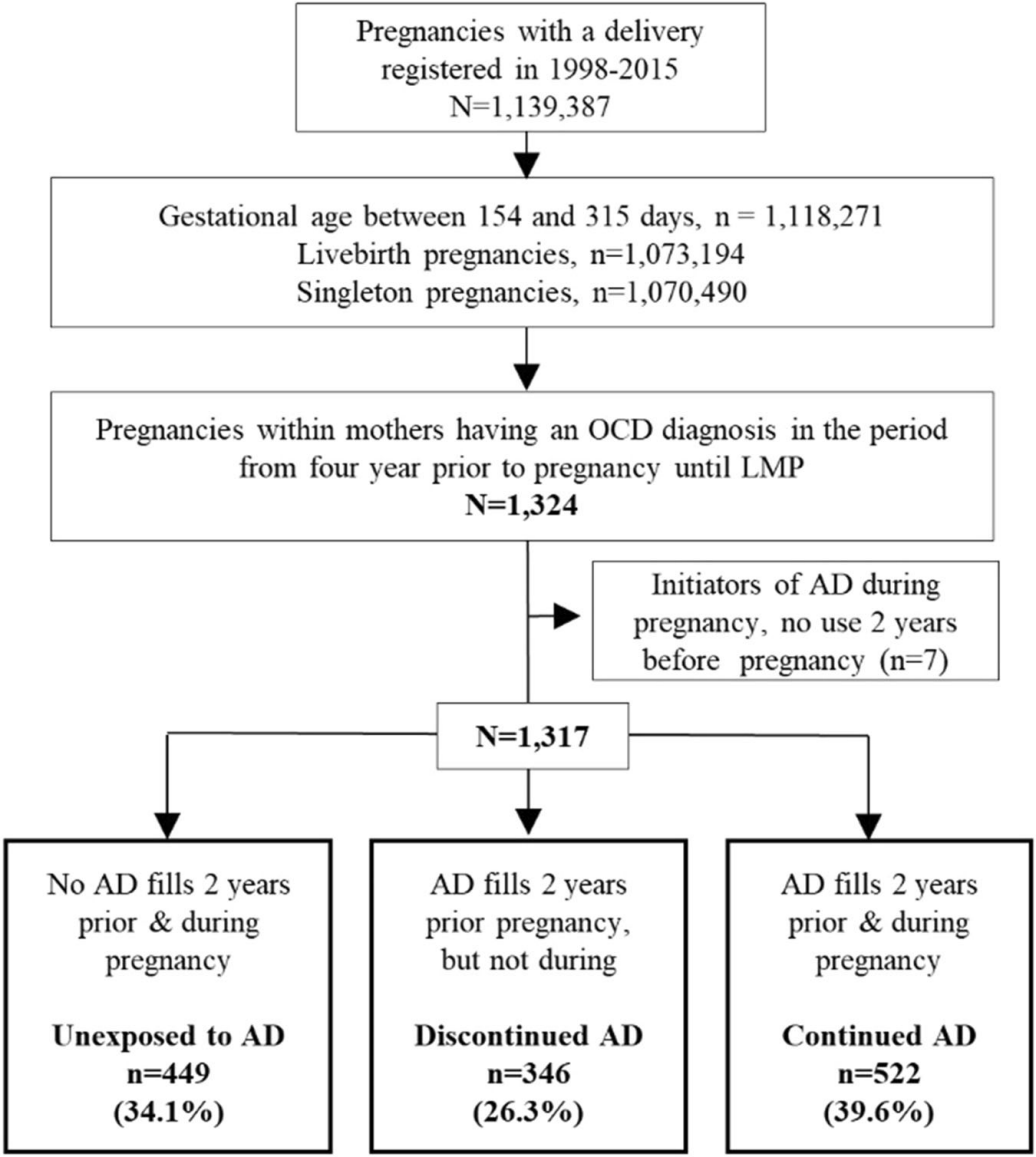
Flowchart to achieve the final study population. AD antidepressant. LMP Last menstrual period. OCD Obsessive-compulsive disorder.

We included liveborn singleton pregnancies among women having fulfilled the following criteria: (1) delivery between 1998 and 2015; (2) gestational age between 154 and 315 days; and (3) having at least one outpatient or inpatient visit with OCD (ICD-10 code: F42) recorded in the National Patient Registry and/or Psychiatric Central Research Register within 4 years prior to last menstrual period date (LMP). The LMP was estimated by subtracting gestational age (primarily based on ultrasound) from the date of birth. The data flow to reach the final study population is shown in Fig. 1.

### Antidepressant exposure

We captured antidepressant exposure using antidepressant prescription fills in the period spanning from 2 years prior to pregnancy to delivery. If a pregnant woman filled at least one antidepressant (Anatomical Therapeutic Chemical [ATC] code N06A) prescription in the 2 years prior to pregnancy with dispensed Defined Daily Doses (DDDs) not overlapping the LMP, she is considered exposed to antidepressants before pregnancy. If a woman filled at least one antidepressant prescription either in the period between LMP and date of birth or in the 30 days prior to pregnancy with dispensed DDDs overlapping the pregnancy window, she is considered exposed to antidepressants during pregnancy. The 30-day window prior to LMP was chosen based on a prior validation study [18]. The sensitivity and specificity of antidepressant prescription fill in pregnancy versus self-report are moderate-high in Scandinavian countries [18, 19]. Antidepressants were sub-divided into selective serotonin reuptake inhibitors (SSRIs, ATC code N06AB), serotonin–norepinephrine reuptake inhibitors (SNRIs, ATC code N06AX), and others (i.e., antidepressants other than SSRIs or SNRIs).

Based on antidepressant exposure status before and during pregnancy, we classified the study population into the following groups: (1) antidepressant continuers in pregnancy (*n* = 522), including pregnancies exposed to antidepressants both before and during pregnancy; (2) discontinuers (*n* = 346), including pregnancies exposed to antidepressant before pregnancy only; (3) unexposed (*n* = 449), including pregnancies with no antidepressant exposure in the studied time window; and (4) initiators (*n* = 7), including women who were exposed to antidepressant during pregnancy only. Given the low number of initiators, these 7 women were excluded from further analyses and descriptions.

### Outcome measures

Our outcome measures were: (1) postpartum episode of OCD, defined as at least one outpatient or inpatient specialist visit with a registered diagnosis of OCD (ICD-10 code: F42) in the postpartum year; and (2) postpartum episode of mood/anxiety disorders, defined as at least one outpatient or inpatient specialist visit with a registered ICD-10 code diagnosis F30-F39 or F40-F41. We included the specific diagnoses of mood/anxiety disorders, given the high comorbidity of OCD with these disorders [13].

### Measured confounders

We considered a set of potential time-fixed confounding factors identified through a literature review and directed acyclic graphs, which included maternal mental health in the mother as binary variables (i.e., familial psychiatric history in the 4 years prior to LMP, hospitalization for psychiatric conditions in the 4 years prior to LMP to delivery; ICD-10: F codes except F42), comorbidities (i.e., asthma, diabetes, epilepsy, hypothyroidism, renal disease), maternal sociodemographic variables (i.e., maternal age, employment status, income, and educational attainment), life-style correlates (e.g., family type, urbanicity at the time of delivery, smoking in pregnancy, antidepressant use of the partner), and pregnancy information (i.e., parity, fertility treatment, congenital anomalies).

Use of other medications, including benzodiazepine/z-hypnotics (ATC codes N05BA, N05CD, N05CF), antipsychotics (ATC code N05A), antiepileptics (ATC code N03A) and opioid analgesics (ATC code N02A) in the first trimester (i.e., first 90 days after LMP), was modelled as time-fixed binary variables (yes/no).

In total, 14.2% of our studied population had missing data on one or more confounders. We imputed missing data on covariates using multiple imputations with chained equations (15 imputations with 20 iterations each), using “*mice*” package [20–23]. Exposure and outcome variables, baseline hazard of the outcome, maternal factors, and auxiliary variables were included in the imputation model.

### Statistical analysis

Characteristics of the study population including maternal sociodemographic, maternal mental health and mental health of the partner and child are descriptively analysed by antidepressant exposure groups.

We estimated the association between antidepressant continuation in pregnancy and postpartum outcomes in the form of hazard ratios (HRs) using unadjusted and weighted Cox regression models with robust standard errors, using time in days since delivery as the time scale. The follow-up period started at birth and ended on the date of the first event, 1 year postpartum, or emigration, whichever came first. We included unadjusted and weighted cumulative event curves for both outcomes [24]. Weighting was done via the inverse probability of treatment weighting (IPTW) method based on the propensity score [25]. The probabilities of “antidepressant *continuers*” relative to “*discontinuers*” or “*unexposed*” were estimated using logistic regression models, given the set of confounders.

Results are shown as unadjusted and weighted HRs with corresponding 95% confidence intervals (CIs). All analyses and plots were conducted using R version 4.0.4 and R studio version 1.4.1106. Balance in the distribution of covariates before and after IPTW was checked via the “*cobalt*” package [26].

### Sensitivity analysis

First, to better understand the role of duration of antidepressant treatment in pregnancy and to minimize the risk of misclassifying discontinuers as continuers, we adopted a stricter definition of antidepressant continuation which requires at least two antidepressant prescription fills during pregnancy (*n* = 424) as continuers, while pregnancies in women filling only one antidepressant prescription in pregnancy were grouped separately (*n* = 98). Second, including multiple pregnancies of the same woman in the cohort may create non-independent observations. We repeated the analyses in the same cohort of women but restricted to first pregnancy in the cohort (*n* = 685). Third, because visits with OCD in the postpartum year can be either follow-up of an episode during pregnancy or a relapse of the disease, we restricted the analysis to those pregnancies with no contact (both in and outpatient) for OCD registered during pregnancy. Fourth, continuers during pregnancy may opt to discontinue antidepressant treatment in the postpartum period, which may influence the risk of exacerbation of psychiatric outcomes. We conducted an additional comparison between antidepressant continuers with discontinuers, stratifying on whether the women had an antidepressant prescription filled in the first three months postpartum. Only participants without the event of interest before the first postpartum prescription fill were considered as having an antidepressant prescription fill. Fifth, we also stratified the analyses by whether the women had inpatient OCD visit prior to birth as a proxy of disease severity.

Data management, analyses and visualization (e.g., random forest plots) were performed using the statistical software R (version 4.1.3).

### Ethical considerations

The study was approved by the Danish Data Protection Agency. No informed consent is required for register-based studies on the basis of anonymized data in accordance with the legislation in Denmark.

## Results

Out of 1,139,387 births between 1998 and 2015 in Denmark, we included 1317 pregnancies within 1126 women (Fig. 1). Of these included pregnancies, 522 (39.6%) continued their antidepressant treatment during pregnancy, 346 (26.3%) discontinued antidepressants before pregnancy, and 449 (34.1%) were unexposed before and during pregnancy. The main characteristics of the study population by antidepressant exposure groups are summarized in Table 1. Compared with unexposed and continuers, discontinuers were more often primiparous, born outside Denmark, smoked during pregnancy and at the time of delivery were outside labour force, had lower educational and lower income, lived in small/medium-size municipalities, and had more often partners with psychiatric history. Antidepressant continuers were characterized by a greater extent of psychiatric history and inpatient psychiatric treatment in the 4 years prior to pregnancy, and used more often other psychotropic drugs compared with discontinuers and especially unexposed. The distribution of confounders between the exposure groups was well-balanced (most standardized mean differences <0.1) after weighting (Figure S1).

**Table 1:** Characteristics of the study population by antidepressant status before and during pregnancy.

	Unexposed to AD (*n* = 449)	AD discontinuers (*n* = 346)	AD continuers (*n* = 522)
*Maternal sociodemographic*			
Age at conception (years); mean (sd)	28.6 (5.2)	27.5 (5.2)	28.8 (5.0)
Primiparity; *n* (%)	205 (45.7)	207 (59.8)	266 (51.0)
Missing	na	na	10 (1.9)
Marital status; *n* (%)			
Married or cohabiting	331 (73.7)	239 (69.1)	370 (70.9)
Not married or cohabiting or missing	118 (26.3)	107 (30.9)	152 (29.1)
Attained educational level^a^; *n* (%)			
Lower than University/College or missing	306 (68.2)	265 (76.6)	377 (72.2)
University/College	143 (31.8)	81 (23.4)	145 (27.8)
Maternal income quintile^a^; *n* (%)			
Quintile 1 or missing	47 (10.4)	44 (12.7)	55 (10.5)
Quintile 2	139 (31.0)	112 (32.4)	131 (25.1)
Quintile 3	118 (26.3)	104 (30.1)	168 (32.2)
Quintile 4	91 (20.3)	60 (17.3)	117 (22.4)
Quintile 5	54 (12.0)	26 (7.5)	51 (9.8)
Occupation^a^			
Employed	202 (45.0)	137 (39.6)	244 (46.7)
Unemployed or missing	10 (2.2)	5 (1.4)	16 (3.1)
Outside labour force	139 (31.0)	138 (40.0)	168 (32.2)
Student	98 (21.8)	66 (19.1)	94 (18.0)
Smoking during pregnancy (yes); *n* (%)	71 (15.8)	87 (25.1)	124 (23.8)
Missing	15 (3.3)	6 (1.7)	11 (2.1)
Calendar year of delivery; *n* (%)			
1998–2002	41 (9.1)	36 (10.4)	35 (6.7)
2003–2006	47 (10.5)	41 (11.8)	63 (12.1)
2007–2011	136 (30.3)	94 (27.2)	197 (37.7)
2012–2015	225 (50.1)	175 (50.6)	227 (43.5)
Place of residence at delivery; *n* (%)			
Capital	87 (19.4)	55 (15.9)	97 (18.6)
Suburb of the capital	61 (13.6)	46 (13.3)	55 (10.5)
Municipalities having a town with more than 100,000 inhabitants	74 (16.5)	39 (11.3)	96 (18.4)
Municipalities having a town with between 10,000 and 100,000 inhabitants	116 (25.8)	108 (31.2)	131 (25.1)
Other municipalities in Denmark or missing	111 (24.7)	98 (28.3)	143 (27.4)
Born in Denmark; *n* (%)	413 (92.0)	307 (88.7)	484 (92.7)
*Maternal mental health*			
Maternal psychiatric history excluding OCD^b^ (yes); *n* (%)	176 (39.2)	152 (43.9)	284 (54.4)
Familial psychiatric history^c^ (yes); *n* (%)	132 (29.4)	84 (24.3)	159 (30.5)
Missing	23 (5.1)	23 (6.6)	22 (4.2)
Inpatient psychiatric treatment^d^ (yes); *n* (%)	89 (19.8)	108 (31.2)	191 (36.6)
Outpatient psychiatric treatment^d^ (yes); *n* (%)	433 (96.4)	344 (99.4)	507 (97.1)
Dispensing of other medications in first trimester (yes); *n* (%)			
Benzodiazepines/z-hypnotics	na	5 (1.4)	29 (5.6)
Antipsychotics	na	9 (2.6)	27 (5.2)
Antiepileptics	5 (1.1)	5 (1.4)	18 (3.4)
Opioid analgesics	na	5 (1.4)	8 (1.5)
Medication use during pregnancy; *n* (%)			
SSRI use during pregnancy	–	–	472 (90.4)
SNRI use during pregnancy	–	–	48 (9.2)
Other antidepressants used during pregnancy	–	–	27 (5.2)
Polytherapy during pregnancy	–	–	24 (4.6)
*Mental health of the partner and child characteristics*			
Paternal psychiatric history at delivery (yes); *n* (%)	54 (12.0)	66 (19.1)	75 (14.4)
Missing	27 (6.0)	20 (5.8)	18 (3.4)
Paternal antidepressant use^d^ (yes); *n* (%)	56 (12.5)	33 (9.5)	73 (14.0)
Missing	27 (6.0)	20 (5.8)	18 (3.4)
Infant sex (male); *n* (%)	231 (51.4)	164 (47.4)	266 (51.0)
Gestational age (days); mean (sd)	276.9 (13.6)	277.0 (13.8)	273.8 (12.9)
Preterm birth; *n* (%)	28 (6.2)	23 (6.6)	48 (9.2)

*AD* antidepressants, SSRI selective serotonin reuptake inhibitors, SNRI serotonin–norepinephrine reuptake inhibitors, OCD Obsessive-compulsive disorder, *na* numbers cannot be presented due to too few observations, number of individuals with missing data was regrouped for some characteristics due to low observations.

^a^Measured 2 years before conception.

^b^Indicates history in the 4 years prior to last menstrual period (LMP).

^c^Indicates history any time prior to delivery.

^d^Indicates history in the 4 years prior to LMP to delivery.

### Postpartum OCD

**Fig. 2:**
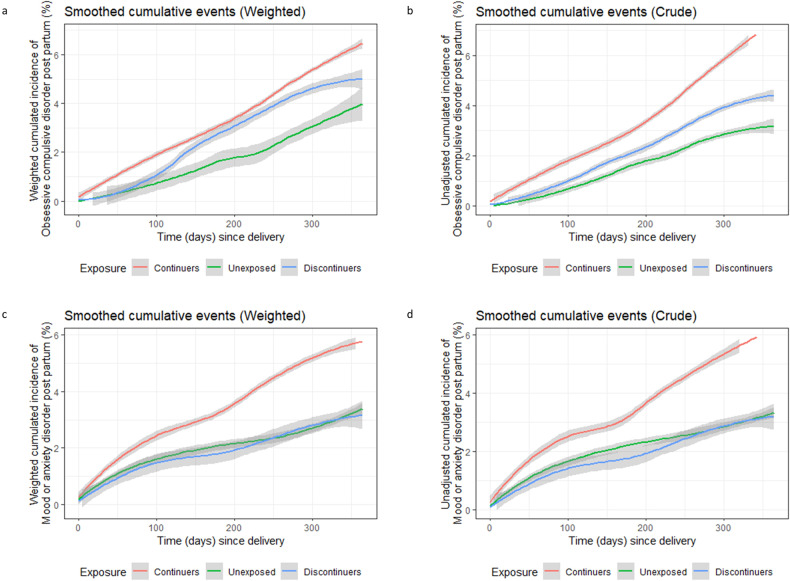
Unadjusted and weighted smoothed cumulative incidence of visits for obsessive compulsive and mood/anxiety disorders in the postpartum year by antidepressant exposure in pregnancy. **a**, **b** Obsessive-compulsive disorder (OCD). **c**, **d** Mood/anxiety disorders.

**Fig. 3:**
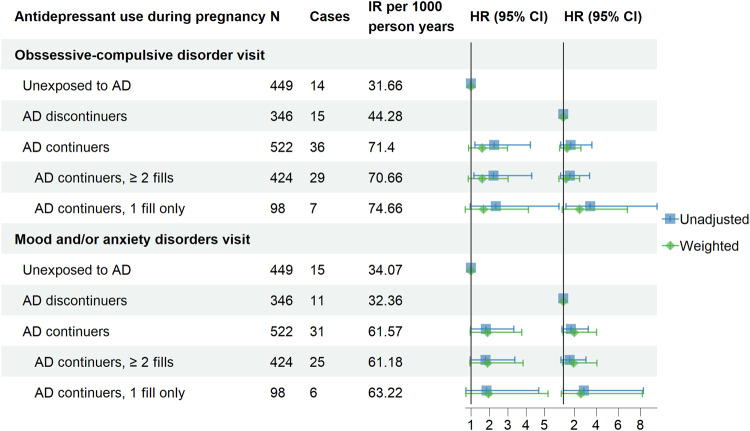
Associations between antidepressant treatment groups and maternal mental health outcomes in the postpartum year among pregnant women with pre-existing obsessive-compulsive disorder. AD antidepressant. Unexposed to AD: without antidepressant exposure in the 2 years prior to pregnancy (before) and during pregnancy. AD discontinuers: having antidepressant exposure before pregnancy only. AD continuers: having antidepressant exposure both before and during pregnancy.

Figure 2a, b shows the smoothed cumulative incidence (unadjusted and weighted) of having a postpartum visit for OCD by antidepressant exposure group. The weighted cumulative incidence was lowest in the unexposed group and highest among continuers. Among discontinuers, the cumulative incidence of postpartum visit for OCD increased sharply from the 3rd to 6th month postpartum reaching that of continuers. In the unadjusted analysis, continuers had a 2.26-fold higher hazard (95% CI: 1.21–4.23) of having OCD visit in the postpartum year than unexposed, but this estimate decreased substantially after weighting (Table 2 and Fig. 3). In the weighted analysis, antidepressant continuers had a moderately elevated hazard for OCD visit postpartum, but the confidence interval crossed the null (HR: 1.34, 95% CI: 0.69–2.62) relative to discontinuers before pregnancy; this point estimate decreased to 1.25 (95% CI: 0.63–2.48) with at least two antidepressants filled in pregnancy (Table 2). However, pregnancies with a single filled prescription for antidepressant had elevated weighted HRs compared to unexposed (weighted HR: 3.44, 95% CI: 1.24–9.51), and to discontinuers before pregnancy (weighted HR: 2.49, 95% CI: 0.91–6.83) (Table 2).

**Table 2:** Associations between antidepressant treatment during pregnancy and maternal mental health outcomes in the postpartum year among pregnant women with pre-existing obsessive-compulsive disorder.

Antidepressant use during pregnancy	No	Cases	IR per 1000 person years	Unadjusted HR (95% CI)	Weighted^a^ HR (95% CI)	Unadjusted HR (95% CI)	Weighted HR (95% CI)
*Obssessive-compulsive disorder visit*							
Unexposed to AD^b^	449	14	31.66	Reference	Reference	–	–
AD discontinuers^c^	346	15	44.28	–	–	Reference	Reference
AD continuers^d^	522	36	71.40	2.26 (1.21, 4.23)	1.67 (0.78, 3.60)	1.61 (0.87, 2.98)	1.34 (0.69, 2.62)
AD continuers, ≥2 fills^e^	424	29	70.66	2.23 (1.16, 4.30)	1.61 (0.76, 3.38)	1.60 (0.84, 3.03)	1.25 (0.63, 2.48)
AD continuers, 1 fill only	98	7	74.66	2.36 (0.96, 5.80)	3.44 (1.24, 9.51)	1.69 (0.69, 4.12)	2.49 (0.91, 6.83)
*Mood and/or anxiety disorders visit*							
Unexposed to AD	449	15	34.07	Reference	Reference	–	–
AD discontinuers	346	11	32.36	–	–	Reference	Reference
AD continuers	522	31	61.57	1.80 (0.98, 3.33)	1.70 (0.89, 3.26)	1.90 (0.96, 3.77)	1.99 (0.99, 4.03)
AD Continuers, ≥2 fills^e^	424	25	61.18	1.79 (0.95, 3.39)	1.58 (0.81, 3.07)	1.89 (0.93, 3.83)	1.95 (0.94, 4.06)
AD continuers, 1 fill only	98	6	63.22	1.85 (0.73, 4.68)	2.89 (1.01, 8.28)	1.95 (0.73, 5.19)	2.61 (0.83, 8.20)

*AD* antidepressants.

^a^The following variables were used for weighting: maternal age, parity, family type, highest attained education, income quintile, smoking, occupational status, calendar year of delivery, urbanicity at time of delivery, comedications in the first trimester (benzodiazepines, antiepileptics, antipsychotics, opioid analgesics), familial psychiatric history, inpatient psychiatric stays, paternal use of antidepressant.

^b^Without antidepressant exposure in the 2 years prior to pregnancy (before) and during pregnancy.

^c^Having antidepressant exposure before pregnancy only.

^d^Having antidepressant exposure both before and during pregnancy.

^e^Having antidepressant exposure both before and during pregnancy with at least 2 antidepressant fills during pregnancy.

### Postpartum mood/anxiety disorders

Figure 2c, d shows the smoothed cumulative incidence (unadjusted and weighted) of visits with a diagnosis of mood/anxiety disorders in the postpartum year. Antidepressant continuers had highest incidence of visits for mood/anxiety disorders, while the incidence in unexposed and discontinuers was comparable. Similar to postpartum OCD, unadjusted and weighted HRs of having postpartum visit for mood/anxiety disorders were also higher in continuers compared to unexposed (weighted HR: 1.70, 95% CI: 0.89 to 3.26) and discontinuers before pregnancy (weighted HR: 1.99, 95% CI: 0.99 to 4.03). The point estimates were lower in continuers with at least two prescription fills during pregnancy, while pregnancy with only one prescription fill had nearly three times higher risk of having postpartum visit for mood/anxiety disorders compared to unexposed (weighted HR: 2.89, 95% CI: 1.01 to 8.28) and to discontinuers during pregnancy (weighted HR: 2.61, 95% CI: 0.83 to 8.20).

### Sensitivity analyses

**Fig. 4:**
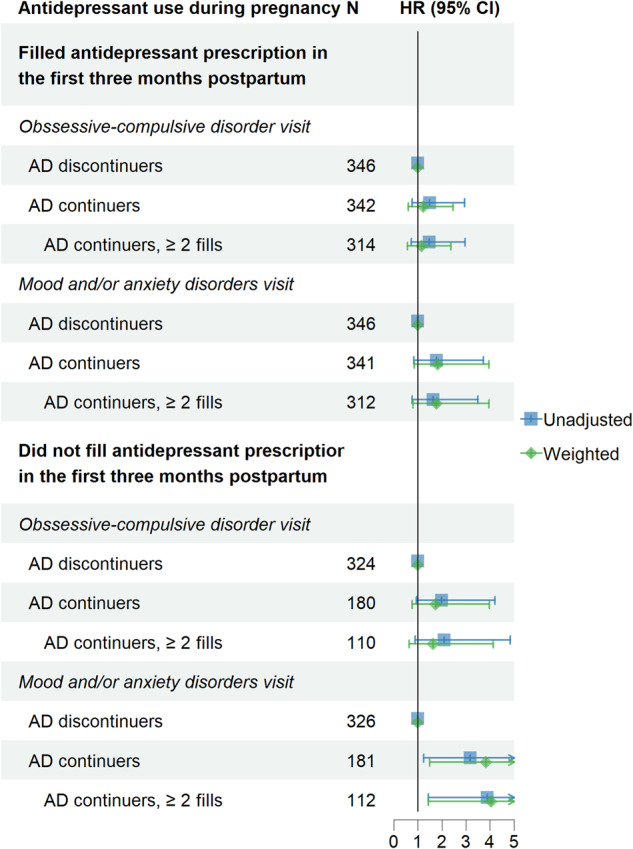
Associations of continued antidepressant in pregnancy with postpartum visits for obsessive-compulsive disorders and mood and/or anxiety disorders, stratified by whether the patient filled antidepressant prescription during the first three months postpartum. AD antidepressant. AD discontinuers: having antidepressant exposure before pregnancy only. AD continuers: having antidepressant exposure both before and during pregnancy.

The results of the various sensitivity analyses (i.e., restricted to first pregnancy and in those without contact for OCD during pregnancy, stratified by having inpatient OCD visit prior to birth) yielded similar results as the main findings (Tables S1–S3, respectively). The stratified analyses (Table S4 and Fig. 4) by whether an antidepressant prescription was filled in three months postpartum revealed higher probability of having a postpartum visit for mood/anxiety disorders in continuers who did not continued antidepressant in early postpartum compared to discontinuers (HR = 3.84, 95% CI: 1.49, 9.92).

## Discussion

Our study investigated the association of antidepressant treatment continuation during pregnancy with postpartum maternal mental health in a national cohort comprising more than 1300 pregnancies within women presenting with pre-existing OCD. About two-third of the study population filled antidepressants in the 2 years prior to pregnancy, with more than half of them maintaining the treatment during pregnancy; of these, two-third continued to fill an antidepressant prescription in the early postpartum. In absolute terms, the probability of having a postpartum visit for OCD and mood/anxiety disorders was 5.9–6.9% for continuers compared to 3.2–4.3% in discontinuers and 3.1–3.3% in unexposed. Our results overall suggest that the likelihood of having postpartum OCD and mood/anxiety disorders visits was not elevated in continuers compared to other treatment groups, including unexposed and discontinuers before pregnancy. However, continuers with only one prescription fill during pregnancy, as well as continuers who did not continue antidepressants in the early postpartum, had significantly increased likelihood of these conditions compared to both discontinuers and unexposed. These findings remained robust across different sensitivity analyses.

The prevalence of antidepressant treatment in our population was nearly 70% before pregnancy and up to 40% during pregnancy. These estimates appear to be higher than in the context of other psychiatric conditions (e.g., depression). This finding may be partly explained by the overestimation of duration of treatment by assuming consumption of one DDD per day [27, 28], while OCD often requires higher dose than that of depression/anxiety which is the common indication of most antidepressants. However, these figures may still suggest that antidepressants play a key role in the treatment of OCD, owing to their demonstrated efficacy and wide availability [29]. On the other hand, treatment discontinuation in pregnancy remains fairly common, as 40% of those on medication before pregnancy did not fill antidepressants anytime during pregnancy, and about 10% filled only one prescription during pregnancy.

We found similar probabilities for postpartum visit for OCD or mood/anxiety disorders in antidepressant continuation compared to treatment discontinuation before pregnancy or unexposed. There are potential explanations for these findings. First, unexposed patients might have milder psychiatric symptoms and do not need to consult for these conditions. Similarly, discontinuation before pregnancy might be clinically justified in some women, and therefore they may be less likely to visit doctors for these conditions. Second, among continuers, there was a subgroup of pregnant women who only filled one prescription during pregnancy, meaning their treatment was either discontinued or interrupted or re-initiated during pregnancy. This subgroup presents nearly three times higher risks of having postpartum OCD and mood/anxiety disorder visits compared to all other treatment groups, especially unexposed. Similarly, the likelihood of having postpartum visit for mood/anxiety disorders in continuers who did not fill antidepressant prescriptions in the early postpartum was nearly four times that of discontinuers.

Our finding suggests that the continuity of treatment during pregnancy and in the postpartum is key in limiting the relapse/onset/continuation of psychiatric conditions in the postpartum period. This is in line with treatment guidelines emphasizing that pharmacotherapy with antidepressants should be maintained for at least a year after therapeutic response, as premature discontinuation of the medication is associated with a high risk of relapse and may blur the treatment response achieved previously [1, 30–32]. Third, in the general non-pregnant population with OCD, higher doses of antidepressants are associated with improved treatment efficacy [33]. In the context of pregnancy, the increased activity of hepatic cytochrome enzymes might require higher dose of antidepressants to archive sufficient treatment response [34]. In addition, the efficacy of some antidepressants could be reduced during pregnancy due to lesser antidepressant concentrations in plasma from increased clearance and greater distribution volume or the interplay between hormonal changes and serotonin availability [35, 36]. However, increasing the dose of antidepressants during pregnancy might not be a common practice given possible dose-dependent risks and concerns about side effects on the unborn child and the mother. It is possible that the continuers in our population were less likely to receive the optimal antidepressant dose during the perinatal period. Our findings, therefore, suggest the need of complementary treatment together with antidepressants during pregnancy to prevent postpartum episodes of mental illnesses. In addition, the decision-making process should involve a detailed psychiatric assessment with many other factors such as individual and family history of the disease, current therapeutic response, level of impairements of different life aspects.

Besides our main findings, we also observed that discontinuers in our population tend to have other sociodemographic characteristics (e.g., employment status, education) and living conditions (e.g., living with partners with psychiatric history) compared to other treatment groups. Identifying factors associated with clinically unjustified antidepressant discontinuation is crucial in treatment counselling, especially in pregnant women. More studies on this topic are needed. In addition, the likelihood of having a postpartum visit for mood/anxiety disorders seems to be higher among first pregnancies. This observation could be a useful consideration in further risk stratification studies.

### Strengths and limitations

Our study relied on population-based data consistently collected over nearly two decades and explores a novel and understudied topic (i.e., antidepressant continuation during pregnancy and postpartum mental health in the context of OCD) in a population comprising more than 1300 pregnancies. Our study design captured pregnancies within women having pre-existing OCD, which helps minimize confounding by indication and allows comparisons with fairer comparator group (i.e., unexposed). In addition, we applied inverse probability treatment weighting using propensity score covering a wide range of sociodemographic and clinical factors, which effectively minimized the risk of measured confounding.

Our study presents with some limitations. First, our study was based on registry-linked data and detailed information on especially dosage, is not captured. As mentioned above, this limitation precludes us from identifying groups with optimum dose of antidepressant treatment. Second, information on precise indication of antidepressants as well as use of psychotherapy was not available in our dataset. Third, we are unable to determine the potential impact of the treatment adherence at baseline and during pregnancy using dispensing data as information regarding adherence (or its proxies) is not recorded in the Danish National Prescription Registry. Although the total number of dispensed defined daily doses are available, measuring adherence based on this information for OCD is not applicable because OCD often requires higher prescribed dose of antidepressant than other conditions (e.g., depression). Fourth, the definition of antidepressant exposure groups based solely on dispensing data might be subjected to misclassification bias, with those with unstable patterns of use being classified among continuers. However, our results remained robust when a stricter definition of continuers with at least two antidepressants was adopted. To address the potential limitation of misclassification, future studies with larger sample sizes and more data available should also consider comparing the risk of postpartum outcomes in women with a single antidepressant prescription versus those with at least two prescriptions in pregnancy. Fifth, symptom severity of OCD conditions and postpartum mental health outcomes were not recorded, leading to residual confounding. Sixth, while our study cohort appears to be relatively large compared to those in other granular studies on the topic, stratifications by antidepressant classes/treatment modality (e.g., switching, dose adjustment) are not feasible because small effect sizes are less likely to be detected. Seventh, we identified our population and defined outcomes based on outpatient and inpatient diagnoses, which may result in misclassifications between treatment groups and outcomes. As a result, potential overlap between OCD and mood/anxiety disorders cannot be excluded. However, it is less likely that systematic bias in favour of one diagnose over the other is present. Eighth, although sensitivity analyses with different specifications remained consistent with our main findings, some analyses could not be done due to a small sample size (e.g., stratified analyses by whether the women had inpatient OCD visit prior to birth).

## Conclusion

In women with pre-existing OCD, there was no difference in the likelihood of having postpartum visits for OCD and mood and/or anxiety disorders following antidepressant continuation during pregnancy relative to discontinuation before pregnancy or non-exposure. However, the probability of these outcomes was elevated in those pregnancies with a single antidepressant prescription filled in pregnancy and in those with continued antidepressants in pregnancy but not in the early postpartum, relative to discontinuers and unexposed. Although this study is based solely on observational data and alone cannot serve directly as guidance for clinical guidelines, our findings serve as a crucial first step toward increasing the evidence base for pharmacological treatment decisions and potential applications in clinical practices for pregnant women with OCD. If replicated, our findings may suggest a possible benefit of antidepressants on maternal mental health in the context of pregnancy and/or for other psychiatric dimensions postpartum and highlight the importance of continuity of treatment throughout the perinatal period. Future systematic reviews and meta-analyses are needed once a sufficient body of literature has emerged.

## Supplementary information

Supplemental material

## Data Availability

The data in this project were delivered by the registry holders to the researchers as pseudonymized data files. Data are available upon request to the registry holders, provided legal and ethical approvals.
