# Supplementary material for: Antidepressant treatment in pregnancy: a Danish registry linkage study in pregnant women with pre-existing obsessive‐compulsive disorder
Source: Transl Psychiatry. 2023 Jun 23;13:223. doi: 10.1038/s41398-023-02516-0 (PMC10290048; doi:10.1038/s41398-023-02516-0)
Supplement: Supplementary file 1 — Supplemental material [file 41398_2023_2516_MOESM1_ESM.docx]

**Antidepressant treatment in pregnancy: a Danish registry linkage study in pregnant women with pre-existing obsessive‐compulsive disorder**

**Authors**: Nhung TH Trinh, Birgitte Dige Semark, Trine Munk-Olsen, Xiaoqin Liu, Suraj Bahadur Thapa, Zeynep Yilmaz, Liselotte Vogdrup Petersen, Angela Lupattelli

**APPENDICES**

Figure S1: Standardized mean difference in confounding factors before and after inverse probability of treatment weighting (IPTW) in the continued antidepressant group versus discontinued (a standardized mean diffference <0.1 indicates characteristics are satisfactorily balanced).

Table S1. Associations between antidepressant treatment during pregnancy and maternal mental health outcomes in postpartum year in pregnant women having pre-existing obsessive-compulsive disorder (restricted to first pregnancy in the cohort).

Table S2. Associations between antidepressant treatment during pregnancy and maternal mental health outcomes in postpartum year in pregnant women having pre-existing obsessive-compulsive disorder (restricted to pregnancies without contact for this condition during pregnancy).

Table S3. Association between antidepressant treatment during pregnancy and maternal mental health outcomes in postpartum year in pregnant women having pre-existing obsessive-compulsive disorder (stratified by whether the women had inpatient visit for obsessive-compulsive disorder prior to birth)

Table S4. Associations of continued antidepressant in pregnancy with postpartum visits for obsessive-compulsive disorders and mood and/or anxiety disorders, stratified by whether the patient filled antidepressant prescription during the first three months postpartum.

**Figure S1: Standardized mean difference in confounding factors before and after inverse probability of treatment weighting (IPTW) in the continued antidepressant group versus discontinued (a standardized mean diffference <0.1 indicates characteristics are satisfactorily balanced).**


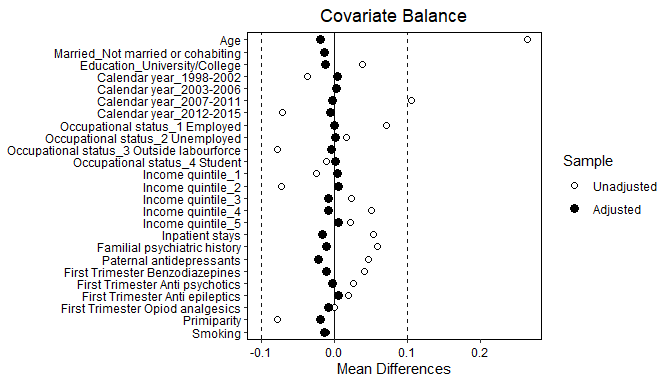


**Table S1. Associations between antidepressant treatment during pregnancy and maternal mental health outcomes in postpartum year in pregnant women having pre-existing obsessive-compulsive disorder (restricted to first pregnancy in the cohort).**

|  | **No** | **Unadjusted HR (95% CI)** | **Weighted HR^*^ (95% CI)** | **Unadjusted HR (95% CI)** | **Weighted HR (95% CI)** |
| --- | --- | --- | --- | --- | --- |
| **Visit for obsessive-compulsive disorder** | | | | | |
| Unexposed^a^ | 206 | Reference | Reference | - | - |
| Discontinuers^b^ | 208 | - | - | Reference | Reference |
| Continuers^c^ | 271 | 1.73 (0.79, 3.82) | 1.81 (0.81, 4.07) | 1.98 (0.87, 4.50) | 2.05 (0.89, 4.75) |
| AD continuers, ≥2 fills ^d^ | 226 | 1.56 (0.68, 3.58) | 1.55 (0.67, 3.62) | 1.79 (0.76, 4.22) | 1.95 (0.72, 4.24) |
| AD continuers, 1 fill only | 45 | 2.61 (0.87, 7.76) | 3.33 (0.90; 12.29) | 2.97 (0.97; 9.07) | 4.19 (1.01, 17.40) |
| **Visit for mood and/or anxiety disorders** | | | | | |
| Unexposed | 206 | Reference | Reference | - | - |
| Discontinuers | 208 | - | - | Reference | Reference |
| Continuers | 271 | 2.45 (0.98, 6.13) | 2.40 (0.94, 6.15) | 2.12 (0.89, 5.03) | 2.04 (0.84, 4.98) |
| AD continuers, ≥2 fills | 226 | 2.64 (1.04, 6.69) | 2.58 (1.00, 6.68) | 2.28 (0.95, 5.49) | 2.45 (0.98, 6.13) |
| AD continuers, 1 fill only | 45 | 1.51 (0.31; 7.45) | 2.91 (0.45, 18.69) | 1.30 (0.27, 6.22) | 2.67 (0.41; 17.58) |

^a^without antidepressant exposure in the two years prior to pregnancy (before) and during pregnancy

^b^having antidepressant exposure before pregnancy only

^c^having antidepressant exposure both before and during pregnancy

^d^having antidepressant exoposure both before and during pregnancy with at least 2 antidepressant fills during pregnancy

^*^ The following variables were used for weighting: maternal age, parity, family type, highest attained education, income quintile, smoking, occupational status, calendar year of delivery, urbanicity at time of delivery, comedications in the first trimester (benzodiazepines, antiepileptics, antipsychotics, opioid analgesics), familial psychiatric history, inpatient psychiatric stays, paternal use of antidepressant.

**Abbreviations: AD=Antidepressants**

**Table S2. Associations between antidepressant treatment during pregnancy and maternal mental health outcomes in postpartum year in pregnant women having pre-existing obsessive-compulsive disorder (restricted to pregnancies without contact for this condition during pregnancy).**

| **Antidepressant use during pregnancy** | **No** | **Unadjusted HR (95% CI)** | **Weighted HR^*^ (95% CI)** | **Unadjusted HR (95% CI)** | **Weighted HR (95% CI)** |
| --- | --- | --- | --- | --- | --- |
| **Visit for obsessive-compulsive disorder** | | | | | |
| Unexposed to AD^a^ | 409 | Reference | Reference | - | - |
| AD Discontinuers^b^ | 300 | - | - | Reference | Reference |
| AD Continuers^c^ | 407 | 1.92 (0.84, 4.36) | 1.39 (0.55, 3.47) | 1.81 (0.74, 4.41) | 1.59 (0.61, 4.16) |
| AD continuers, ≥2 fills ^d^ | 263 | 2.09 (0.88, 4.97) | 1.65 (0.66, 4.11) | 1.97 (0.78, 5.01) | 1.51 (0.53, 4.30) |
| AD continuers, 1 fill only | 77 | 1.81 (0.49; 6.75) | 1.80 (0.43; 7.54) | 1.71 (0.44; 6.62) | 1.35 (0.34; 5.36) |

^a^without antidepressant exposure in the two years prior to pregnancy (before) and during pregnancy

^b^having antidepressant exposure before pregnancy only

^c^having antidepressant exposure both before and during pregnancy

^d^having antidepressant exoposure both before and during pregnancy with at least 2 antidepressant fills during pregnancy

^*^ The following variables were used for weighting: maternal age, parity, family type, highest attained education, income quintile, smoking, occupational status, calendar year of delivery, urbanicity at time of delivery, comedications in the first trimester (benzodiazepines, antiepileptics, antipsychotics, opioid analgesics), familial psychiatric history, inpatient psychiatric stays, paternal use of antidepressant.

**Abbreviations: AD=Antidepressants**

**Table S3. Association between antidepressant treatment during pregnancy and maternal mental health outcomes in postpartum year in pregnant women having pre-existing obsessive-compulsive disorder (stratified by whether the women had inpatient visit for obsessive-compulsive disorder prior to birth)**

| **Antidepressant use during pregnancy** | **No** |  | **Unadjusted**  **HR (95% CI)** | **Weighted**†  **HR (95% CI)** |
| --- | --- | --- | --- | --- |
| **Had inpatient visit for obsessive-compulsive disorder prior to birth** | | | | |
|  | **Visit for obsessive-compulsive disorder** | | | |
| AD discontinuers^a^ | 108 |  | Reference | Reference |
| AD continuers^b^ | 191 |  | 2.22 (0.83, 5.94) | 1.73 (0.55, 5.38) |
| AD continuers, ≥ 2 fillings^c^ | 154 |  | 2.33 (0.85, 6.39) | 1.72 (0.55, 5.37) |
|  | **Mood and/or anxiety disorder visit** | | | |
| AD discontinuers^a^ | 108 |  | Reference | Reference |
| AD continuers^b^ | 191 |  | 1.48 (0.52, 4.17) | 1.80 (0.62, 5.20) |
| AD continuers, ≥ 2 fillings^c^ | 154 |  | 1.70 (0.60, 4.86) | 2.17 (0.73, 6.44) |
| **No inpatient visit for obsessive-compulsive disorder prior to birth** | | | | |
|  | **Visit for obsessive-compulsive disorder** | | | |
| AD discontinuers^a^ | 238 |  | Reference | Reference |
| AD continuers^b^ | 331 |  | 1.23 (0.56, 2.67) | 1.09 (0.49, 2.43) |
| AD continuers, ≥ 2 fillings^c^ | 270 |  | 1.14 (0.50, 2.60) | 0.95 (0.41, 2.19) |
|  | **Mood and/or anxiety disorder visit** | | | |
| AD discontinuers^a^ | 238 |  | Reference | Reference |
| AD continuers^b^ | 331 |  | 2.20 (0.88, 5.53) | 1.80 (0.62, 5.20) |
| AD continuers, ≥ 2 fillings^c^ | 270 |  | 1.95 (0.74, 5.10) | 2.17 (0.73, 6.44) |

^a^having antidepressant exposure before pregnancy only

^b^having antidepressant exposure both before and during pregnancy

^c^having antidepressant exoposure both before and during pregnancy with at least 2 antidepressant fills during pregnancy

†The following variables were used for weighting: maternal age, parity, family type, highest attained education, income quintile, smoking, occupational status, calendar year of delivery, urbanicity at time of delivery, comedications in the first trimester (benzodiazepines, antiepileptics, antipsychotics, opioid analgesics), familial psychiatric history, inpatient psychiatric stays, paternal use of antidepressant.

**Abbreviations: AD=Antidepressants**

**Table S4. Associations of continued antidepressant in pregnancy with postpartum visits for obsessive-compulsive disorders and mood and/or anxiety disorders, stratified by whether the patient filled antidepressant prescription during the first three months postpartum.**

| **Antidepressant use during pregnancy** | **No** |  | **Unadjusted**  **HR (95% CI)** | **Weighted**†  **HR (95% CI)** |
| --- | --- | --- | --- | --- |
| **Filled antidepressant prescription in the first three months postpartum** | | | | |
|  | **Visit for obsessive-compulsive disorder** | | | |
| AD discontinuers^a^ | 346 |  | Reference | Reference |
| AD continuers^b^ | 342 |  | 1.50 (0.76, 2.95) | 1.23 (0.61; 2.47) |
| AD continuers, ≥ 2 fillings^c^ | 314 |  | 1.48 (0.74; 2.97) | 1.16 (0.57, 2.38) |
|  | **Mood and/or anxiety disorder visit** | | | |
| AD discontinuers^a^ | 346 |  | Reference | Reference |
| AD continuers^b^ | 341 |  | 1.77 (0.84, 3.72) | 1.84 (0.85; 3.96) |
| AD continuers, ≥ 2 fillings^c^ | 312 |  | 1.63 (0.76, 3.50) | 1.78 (0.80, 3.96) |
| **Did not fill antidepressant prescription in the first three months postpartum** | | | | |
|  | **Visit for obsessive-compulsive disorder** | | | |
| AD discontinuers^a^ | 324 |  | Reference | Reference |
| AD continuers^b^ | 180 |  | 1.99 (0.94, 4.21) | 1.75 (0.77, 3.97) |
| AD continuers, ≥ 2 fillings^c^ | 110 |  | 2.09 (0.90, 4.84) | 1.63 (0.64, 4.14) |
|  | **Mood and/or anxiety disorder visit** | | | |
| AD discontinuers^a^ | 326 |  | Reference | Reference |
| AD continuers^b^ | 181 |  | 3.17 (1.25, 8.05) | 3.84 (1.49, 9.92) |
| AD continuers, ≥ 2 fillings^c^ | 112 |  | 3.89 (1.45; 10.44) | 4.04 (1.45, 11.29) |

^a^having antidepressant exposure before pregnancy only

^b^having antidepressant exposure both before and during pregnancy

^c^having antidepressant exoposure both before and during pregnancy with at least 2 antidepressant fills during pregnancy

†The following variables were used for weighting: maternal age, parity, family type, highest attained education, income quintile, smoking, occupational status, calendar year of delivery, urbanicity at time of delivery, comedications in the first trimester (benzodiazepines, antiepileptics, antipsychotics, opioid analgesics), familial psychiatric history, inpatient psychiatric stays, paternal use of antidepressant.

**Abbreviations: AD=Antidepressants**
